# Suprascapular Chest Tube Placement for Symptomatic Spontaneous Pneumothorax: The Forgotten Access Point

**DOI:** 10.1002/rcr2.70644

**Published:** 2026-07-15

**Authors:** Sammy Onyancha, Bardia Amirmiran, Tanja Dieball, Pavel Eliseev, Gernot Rohde

**Affiliations:** ^1^ Department of Pulmonology St. Elisabethen Krankenhaus Frankfurt Germany; ^2^ Department of Respiratory Medicine Universitätsklinikum Marburg Marburg Germany

**Keywords:** Bülau position, chest drain, chest tube, Monaldi position, pneumothorax, suprascapular approach

## Abstract

Spontaneous pneumothorax is a common complication of advanced interstitial and emphysematous lung disease. Chest tube drainage is the mainstay of pneumothorax management. Conventional insertion sites—lateral placement in the 4th–6th intercostal space (Bülau position) and anterior placement in the 2nd–3rd intercostal space (Monaldi position) are widely used but may be contraindicated in specific clinical scenarios. The suprascapular (posterior apical) approach represents a rarely utilised alternative. We report a 72‐year‐old male with COPD and emphysema who presented with a symptomatic apical pneumothorax. Computed tomography demonstrated minimal lateral pleural separation, making lateral access unsafe. The anterior route was also contraindicated due to a large postoperative wound following recent surgery. A suprascapular approach was therefore selected. Under local anaesthesia, an 8Fr pigtail catheter was inserted using anatomical landmarks and the pneumothorax was successfully evacuated with continuous negative pressure without complications. This case demonstrates that suprascapular chest tube placement can be a safe and effective alternative in selected patients with apical pneumothorax when standard insertion sites are contraindicated.

## Graphical abstract

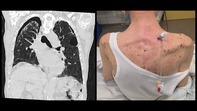

## 1 Introduction

Chest tube drainage is central to pneumothorax management in interventional pulmonology. The most common insertion sites are:

- **Lateral placement (‘Bülau position’)**: First described by Gotthard Bülau, a German pulmonologist who used this position for chest drains as treatment for pleural empyema, typically involves insertion in the mid‐axillary line between the 4th and 6th intercostal spaces.
- **Anterior placement (‘Monaldi position’)** Described by Vincenzo Monaldi, an Italian physician who used this position for chest drains as part of tuberculosis treatment, typically involves insertion at the mid‐clavicular line between the 2nd and 3rd intercostal space.

These sites are generally effective and safe but may not always be feasible. Anatomical variations, obesity, chest wall deformities, prior thoracic surgery, indwelling devices (such as pacemakers or defibrillators) and postoperative wounds can render conventional access hazardous or technically challenging. In such situations, alternative approaches to the pleural space must be considered to avoid delays in decompression and to minimise the risk of iatrogenic complications.

One such alternative is the suprascapular (posterior apical) approach. This technique provides direct access to the apical pleural space and was described in earlier surgical and interventional reports as a safe and effective means of draining apical pneumothoraces. Although historically recognised, its use has largely declined in modern practice and it is rarely mentioned in contemporary guidelines. This lack of visibility may lead to underutilisation, particularly in complex clinical cases where it could represent the most suitable route.

Given the increasing prevalence of patients with conditions associated with higher rates of spontaneous pneumothorax, such as bullous emphysema or advanced interstitial lung diseases, there is a growing need for flexible and individualised management strategies. In this context, revisiting and re‐evaluating rarely used access techniques such as the suprascapular approach is clinically relevant.

We describe the case of a 72‐year‐old male with COPD and emphysema who developed an apical pneumothorax in whom both conventional chest tube placements were contraindicated and who was successfully treated via the suprascapular approach.

## 2 Case Report

A 72‐year‐old male with chronic obstructive pulmonary disease (COPD) and advanced emphysema presented with acute dyspnoea and chest pain. His medical history included multiple hospitalisations for COPD exacerbations and ongoing treatment with inhaled bronchodilators.

**FIGURE 1:**
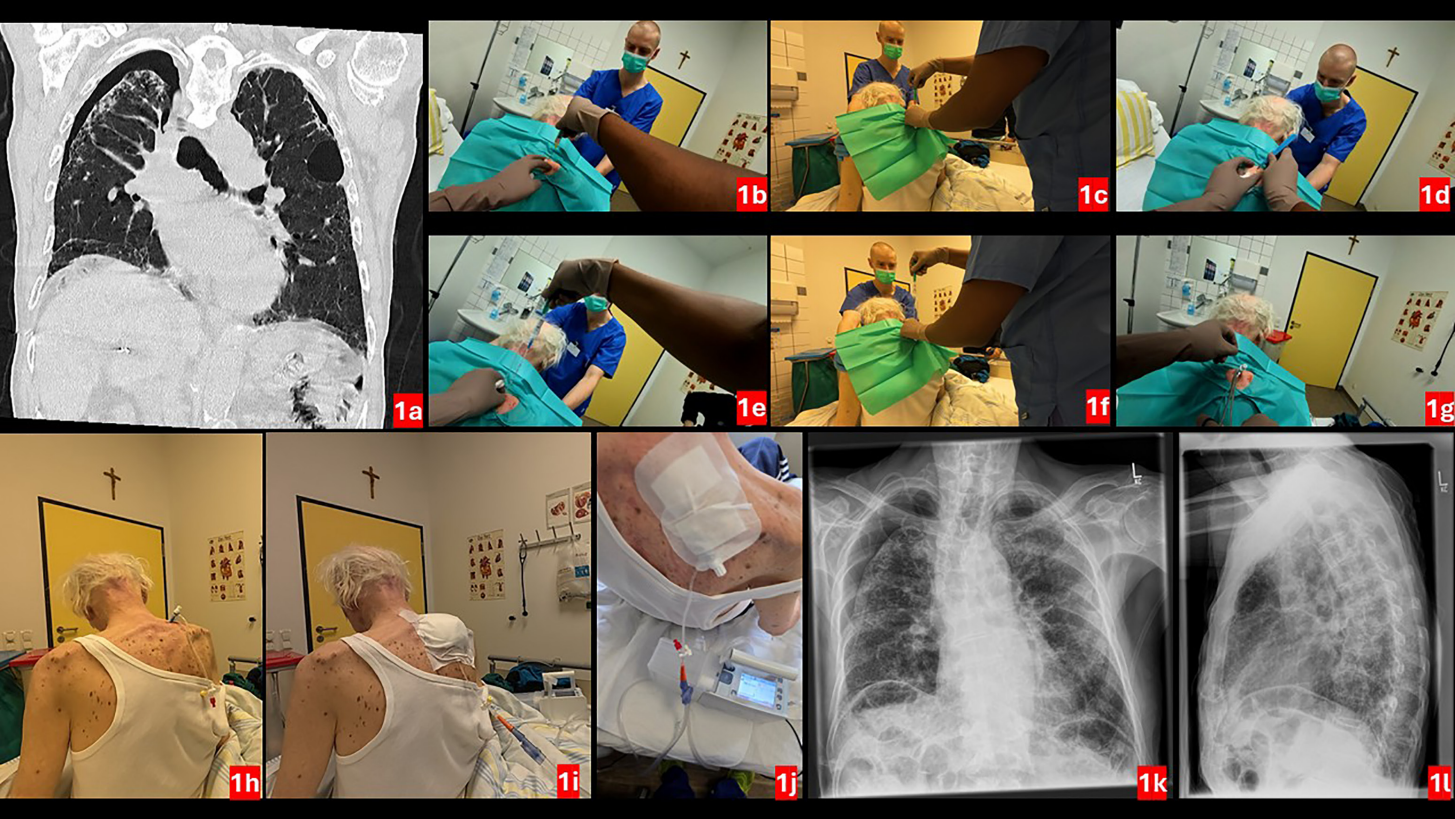
(a) Initial CT‐scan showing apical pneumothorax. (b, c) Local anaesthetic infiltration. (d) Skin incision before chest tube insertion. (e, f) Insertion of chest drain with trocar. (g) Chest drain suture placement. (h–j) Attachment to portable electronic chest drainage system. (k, l) Chest x‐ray showing apical placement of chest tube.

He was mildly tachypnoeic with an oxygen saturation of 90% on room air. Laboratory investigations were unremarkable. Chest computed tomography (CT) revealed a right apical pneumothorax with minimal lateral separation between the visceral and parietal pleura (Figure 1a). The lack of lateral pleural space rendered conventional lateral insertion unsafe due to a high risk of iatrogenic lung injury. Moreover, the anterior route was not feasible, as the patient had recently undergone surgery with a large healing wound in the 2nd intercostal space.

Given these limitations, a suprascapular approach was chosen. After aseptic preparation, infiltration of local anaesthesia was performed with 10 mL of xylocaine paravertebrally at the midline between the spinous process and the superior angle of the scapula (Figure 1b,c). The needle was then progressively inserted perpendicularly to the skin under continuous aspiration. Aspiration of air confirmed entry into the void caused by the pneumothorax. The needle was withdrawn under continuous infiltration with anaesthesia to numb the tract. After infiltration, a small skin incision was made (Figure 1d) and an 8Fr Pigtail Catheter (Safe‐T‐Centesis, ewimed GmbH, Hechingen‐Boll, Germany) was inserted using a Veres trocar system into the apical pleural space (Figure 1e,f). The trocar was removed and air was aspirated through the pigtail catheter to confirm its proper placement within the pneumothorax. The catheter was then sutured on (Figure 1g) and connected to a portable electronic chest drainage system (Thopaz+, Medela Inc., Baar, Switzerland) (Figure 1h–j). Finally, a continuous negative pressure of −15cm H_2_O was applied resulting in immediate symptomatic improvement (Video 1). Post‐procedural imaging confirmed proper placement and progressive re‐expansion of the lung (Figure 1k,l). The patient remained stable with no evidence of new air leaks, bleeding or catheter malposition. The chest drain remained in situ for 4 days, after which it was successfully removed.

**VIDEO 1:** Step by step—suprascapular chest drain placement. Video content can be viewed at https://onlinelibrary.wiley.com/doi/10.1002/rcr2.70644.

## 3 Discussion

This case highlights the clinical utility of the suprascapular approach for chest tube placement, particularly in complex or anatomically challenging cases. While the Bülau and Monaldi positions remain the gold standards for chest tube placement [1, 2], they may not always be feasible due to altered anatomy, surgical wounds, obesity, chest wall deformities or the presence of implanted devices. In such scenarios, awareness of the less commonly used suprascapular approach can be lifesaving.

The suprascapular approach provides direct access to the apical pleural space, making it particularly useful in cases of localised apical pneumothorax [3]. Historically, reports from the 1970s to 1990s described the feasibility of posterior apical drainage [4, 5, 6], but its use has largely declined in the modern era, possibly due to the standardisation of lateral and anterior insertions in clinical guidelines.

In cases where conventional chest tube insertion is not feasible, alternative management strategies include video‐assisted thoracoscopic surgery (VATS) or conservative management. VATS provides definitive treatment, allowing for direct visualisation, resection of blebs and pleurodesis. However, it is invasive, requires general anaesthesia and may carry a substantial perioperative risk in elderly patients or those with advanced pulmonary disease, such as COPD or interstitial lung disease. As this case illustrates, reintroducing the suprascapular technique into the repertoire of interventional pulmonologists may help avoid unnecessary surgical interventions in patients where conventional routes are contraindicated.

Conversely, conservative management (oxygen therapy and observation) may be appropriate in small or minimally symptomatic pneumothoraces, but is often insufficient in symptomatic patients or those with compromised respiratory reserve. In the present case, conservative management was not appropriate due to the patient's symptoms and underlying lung disease.

A further advantage of the suprascapular approach is that it allows the use of small‐bore catheters. Small‐bore pigtail drains are increasingly favoured for pneumothorax management, given their comparable efficacy to large‐bore tubes and superior patient comfort. In our case, the use of an 8Fr catheter was sufficient to evacuate the pneumothorax effectively while minimising insertion trauma and reducing the risk of complications such as bleeding and infection.

Potential risks of the suprascapular route include injury to adjacent vascular and neural structures, particularly the subclavian vessels and brachial plexus. These risks highlight the importance of careful anatomical knowledge, the use of imaging guidance and meticulous patient selection. In this case, pre‐procedural computed tomography (CT) was essential for identifying the apical localisation of the pneumothorax and the absence of a safe lateral access window. CT imaging also helped exclude anatomical variants and define a safe trajectory for posterior access. While the procedure in this case was performed using anatomical landmarks, real‐time imaging guidance (such as ultrasound or CT‐guided insertion) could further enhance safety, particularly in less experienced hands or in patients with complex anatomy.

Another aspect worth considering is training and awareness. Because the suprascapular approach is rarely performed, many younger clinicians may not be familiar with the technique. Incorporating it into interventional pulmonology teaching and simulation‐based training could help to ensure that it remains an available option in difficult cases.

Finally, the broader clinical implication of this case is that individualised management strategies are essential in pleural disease. Standardised approaches remain the first‐line choice, but flexibility and awareness of alternative access routes expand the therapeutic options, potentially preventing delays in treatment, procedural complications or the need for more invasive interventions.

## Author Contributions

All the authors contributed to the manuscript. The first draft of the manuscript was written by Sammy Onyancha, and all the authors commented on previous versions of the manuscript. All the authors have read and approved the final manuscript.

## Funding

The authors have nothing to report.

## Consent

The authors declare that written informed consent was obtained for the publication of this manuscript and accompanying images using the form provided by the Journal.

## Conflicts of Interest

The authors declare no conflicts of interest.

## Data Availability

The data that support the findings of this study are available from the corresponding author upon reasonable request.
